# The role of visual processing on tactile suppression

**DOI:** 10.1371/journal.pone.0195396

**Published:** 2018-04-04

**Authors:** Hanna Gertz, Katja Fiehler, Dimitris Voudouris

**Affiliations:** Experimental Psychology, Justus-Liebig University Giessen, Germany; University of Muenster, GERMANY

## Abstract

It has been suggested that tactile signals are suppressed on a moving limb to free capacities for processing other relevant sensory signals. In line with this notion, we recently showed that tactile suppression is indeed stronger in the presence of reach-relevant somatosensory signals. Here we examined whether this effect also generalizes to the processing of additional visual signals during reaching. Brief vibrotactile stimuli were presented on the participants’ right index finger either during right-hand reaching to a previously illuminated target LED, or during rest. Participants had to indicate whether they detected the vibrotactile stimulus or not. The target LED remained off (*tactile*), or was briefly illuminated (*tactile & vis*) during reaching, providing additional reach-relevant visual information about the target position. If tactile suppression frees capacities for *reach-relevant* visual information, suppression should be stronger in the *tactile & vis* compared to the *tactile* condition. In an additional visual-discrimination condition (*tactile & visDis*), the target LED flashed once or twice during reaching and participants had to also report the number of flashes. If tactile suppression occurs to free additional capacities for *perception-relevant* visual signals, tactile suppression should be even stronger in the *tactile & visDis* compared to the *tactile & vis* condition. We found that additional visual signals improved reach endpoint accuracy and precision. In all conditions, reaching led to tactile suppression as indicated by higher detection thresholds compared to rest, confirming previous findings. However, tactile suppression was comparable between conditions arguing against the hypothesis that it frees capacities for processing other relevant visual signals.

## Introduction

It has been suggested that sensorimotor predictions established by forward models influence human perception [1]. For instance, humans suppress the sensations evoked as a consequence of their own actions, either when self-tickling [2, 3] or when producing force [4, 5]. There is evidence that humans also suppress externally applied tactile stimuli presented on a limb shortly before it starts moving or during movement [6–10]. Although postdictive mechanisms may also be involved in tactile suppression [11], there is ample evidence that the generation of a motor plan itself is adequate to attenuate the sensation of afferent peripheral signals. For instance, neuroimaging studies show reduced activity in secondary somatosensory areas related to either externally [12, 13] or self-generated somatosensory signals [14, 15] associated with a movement. Similarly, electrical stimuli presented on a limb planned to move, but is not moving due to a delay in motor execution through TMS, are suppressed presumably due to the generation of the movement plan [7]. Thus, both neurophysiological and behavioral evidence indicate that the sensation of both self-produced and externally generated somatosensory stimuli around a movement is attenuated.

It is proposed that the suppression of somatosensory signals from the moving limb may occur to reduce the signals’ sensory saliency [5], which may facilitate the processing of other sensory input important for the task [1, 16]. In line with this idea, we have shown that the sensitivity to tactile stimuli on the reaching hand is reduced while the sensitivity is in parallel enhanced on the unseen target hand [17]. The reduction of tactile sensitivity, which we will be hereafter referring to as *tactile suppression*, is related to the tactile enhancement, which may facilitate the guidance of the reaching hand to the somatosensory target. In this line, tactile suppression on the reaching hand is stronger when processing somatosensory signals from the unseen target hand than when reaching to an external target [18]. Thus, the stronger suppression on the reaching hand likely occurs to facilitate the processing of the reach-relevant somatosensory signals from the target hand. The strength of tactile suppression is also modulated during grasping, with suppression being weaker on digits that are involved in grasping [10], possibly to facilitate efficient information uptake from these digits. These results support the notion that tactile suppression occurs to facilitate processing of relevant sensory signals [1], at least of signals from the somatosensory system.

It remains unclear whether tactile suppression also occurs to facilitate the processing of relevant signals from sensory modalities other than the somatosensory system. For instance, when performing a visually-guided hand movement, visual signals are particularly important as they are used to improve movement control by leading to more accurate reaching [19] or faster grasping movements [20]. Because of their importance, the processing of visual signals during reaching may be enhanced while signals from other modalities may be suppressed. For instance, there is decreased susceptibility to an audio-visual fusion illusion presented on the movement target during reaching [21]. This result has been explained by enhanced processing of the reach-relevant visual signals [21], which may be related to auditory suppression [22]. In other words, the processing of the task-unrelated auditory signals is deteriorated to prioritize the processing of the task-relevant visual signals [21]. Crucially, such movement-induced dynamic modulation of visual and auditory perception may be transferable to the processing of visual and tactile signals during reaching.

Here, we examined the hypothesis whether tactile suppression occurs to facilitate the processing of reach-relevant and/or perception-relevant visual signals. We consider tactile suppression to be reflected in increased detection thresholds and poorer detection precision. Tactile suppression is mostly represented by weaker perceived stimulus intensity, but the modulation of detection thresholds is often associated with a similar modulation in detection precision [18, 23, 24], therefore we also examined the modulation of the detection precision. Participants sat in darkness and had to detect a brief vibrotactile stimulus on their right index finger presented either in rest or during reaching towards a visual target that was illuminated until movement onset. In the *tactile* condition, participants did not have any on-line visual information about the target position during reaching. In line with previous studies (e.g., [9]), we expect that tactile stimuli will be suppressed during movement compared to rest. In the *tactile & vis* condition, we examined whether tactile suppression facilitates the processing of reach-relevant visual signals. We briefly flashed the previously illuminated target during reaching, providing additional reach-relevant visual information about its position. If participants process these reach-relevant visual signals, their endpoint accuracy and precision should be improved compared to the *tactile* condition. If tactile suppression occurs to facilitate the processing of these reach-relevant visual signals, we expect suppression to be stronger in the *tactile & vis* than the *tactile* condition. Consequently, a release of additional processing capacities should also be reflected in improved movement control. To examine whether tactile suppression occurs to facilitate the processing of additional perception-relevant visual information, we briefly illuminated the target either once or twice during reaching and asked participants to also indicate how many flashes they saw (*tactile & visDis*). The visual information about the number of flashes is relevant for the visual discrimination task, but irrelevant for the reach. If tactile suppression occurs to facilitate the processing of such perception-relevant visual signals, we expect suppression to be stronger in the *tactile & visDis* than the *tactile & vis* condition. Consequently, the visual discrimination performance should be related to the strength of tactile suppression, with improved visual discrimination being related to stronger suppression. However, if the performance of the visual discrimination task requires additional processing capacities that are withdrawn from the tactile system, tactile perception performance should be impaired. Lastly, based on our previous results [18], we hypothesize that the strength of suppression should be related to movement guidance. In this case, we expect greater endpoint accuracy and precision with stronger tactile suppression, independently of whether additional visual signals are presented or not. If the visual discrimination task adds additional processing demands, we might expect a reduction in the movement guidance.

## Materials and methods

### Participants

Twenty-two volunteers participated in this study. Nine participants were excluded either because at least one of their estimated detection thresholds was outside the range of our stimulation or due to unsuccessful kinematic data collection (see *Data analysis* for more details). This resulted in a final sample of 13 participants (18–33 years, mean age: 23.7 years; 11 females), on which all subsequent analyses were based on. Participants were right-handed according to the Edinburgh Handedness Inventory (88.3±18.6; [25]). Prior to the experiment they gave informed written consent, and after the experiment they received course credits or financial compensation for their effort. The study was approved by the local ethics committee of the Justus-Liebig University Giessen and was in accordance with the Declaration of Helsinki (2008).

### Experimental set-up

**Fig 1:**
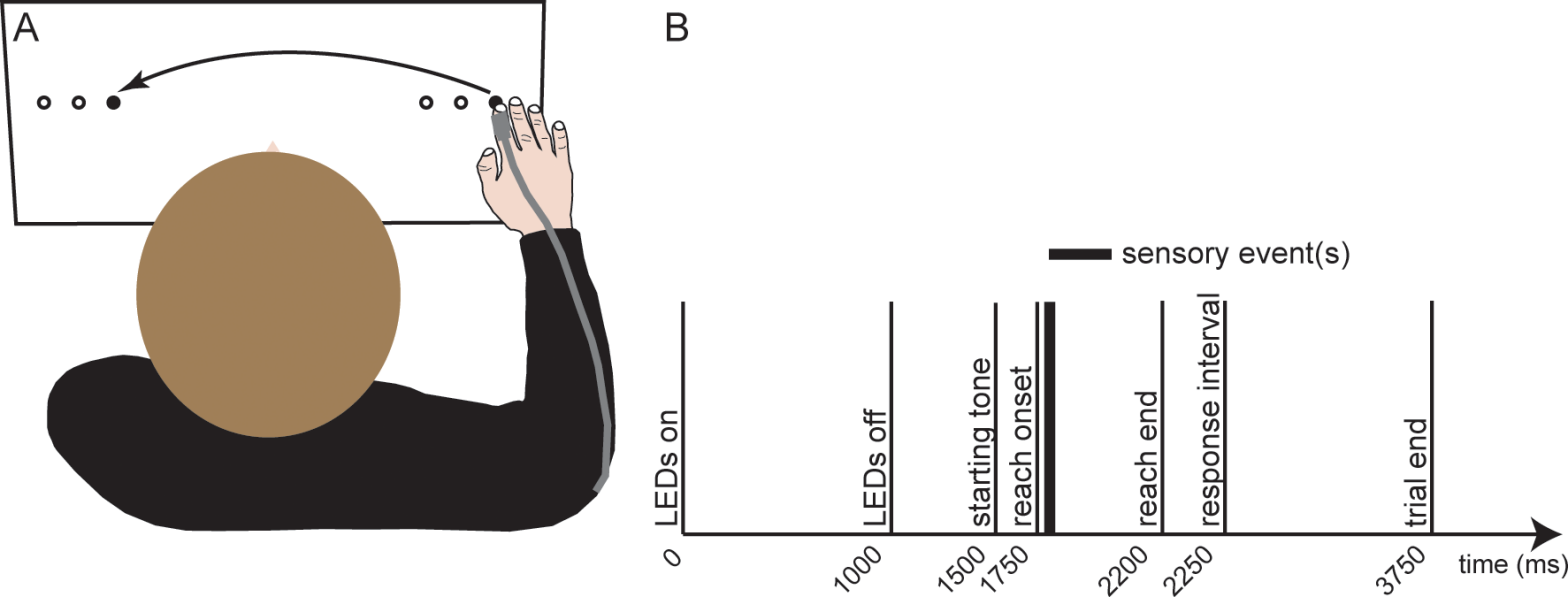
**Illustration of (A) the set-up, and (B) timeline of a reaching trial.** Participants placed their right index finger on the illuminated starting LED and fixated with their eyes the illuminated target LED. After the LEDs turned off, a tone prompted participants to start reaching to the target LED. Right after movement onset, the tactile stimulus was presented on the moving index finger; in the reaching block of the *tactile & vis* condition this was accompanied with the target LED being briefly illuminated once more, and in the reaching block of the *tactile & visDis* condition with the target LED being shortly illuminated either once or twice. After the reaching movement was completed, participants had to indicate whether they felt a tactile stimulus and, in addition, in the *tactile & visDis* condition whether they saw one or two flashes. The starting and the target LEDs were always on the right and left of the participants’ midline, respectively. All possible LED positions are depicted here with circles with one combination highlighted (filled). The same configurations were used also for the baseline block of each condition, with the difference that the participants’ right hand rested on the rightmost part of the touchscreen. The times indicated after the starting tone are approximated as they depended on the latency of the reaching movement.

Participants sat in front of a table in darkness with their head resting on a chin-rest. A transparent touchscreen (430 x 330 x 30 mm; MagicTouch 2.0, Keytec, Inc., Garland, Texas, USA) was placed on the table centrally and slanted 15° with respect to the horizontal plane, with its rear edge elevated, and its centre being ~ 40 cm below the participants’ eyes and 20 cm away from their body (Fig 1). A horizontal board of light emitting diodes (LEDs) was mounted centrally below the touchscreen and spanned across its entire width. Because the experiment was performed in a dark room, the board of LEDs was hardly visible to the participants. Three LEDs served as the starting positions and another three LEDs as target positions for the reaching movement. Starting and target positions were varied to minimize stereotypical movements; yet, they were combined such that the movement amplitude was always constant (22 cm). More specifically, starting positions were at 17 cm, 14 cm, and 11 cm right from the body midline, while the respective target positions were placed at 5 cm, 8 cm, and 11 cm left from the body midline. Auditory signals prompted participants to start reaching and to respond to the tactile detection and, if applicable, to the visual discrimination tasks. Responses to these perceptual tasks were recorded via a keypad placed ~5cm on the left of the touchscreen, where participants kept their left hand during the experiment.

Vibrotactile stimuli (250 Hz, 25 ms) were presented on the dorsal part of the right index finger using a custom-made vibrotactile stimulation device (Engineer Acoustics Inc., Florida, USA). We used 15 stimuli differing in intensity: their peak-to-peak displacement ranged from 0.00316 mm to 0.0948 mm in steps of 0.00632 mm. Reaches were recorded at 100 Hz with an Optotrak Certus (Northern Digital Inc., Waterloo, ON, Canada) that tracked a marker placed on the right index fingernail.

### Procedure

The experiment comprised three conditions (*tactile*, *tactile & vis*, *tactile & visDis*) with each condition consisting of two blocks: a baseline block without movement and a reaching block resulting in a total of six blocks. Baseline blocks were used to quantify the effect of tactile suppression during movement.

In both *tactile* blocks, participants performed only the tactile detection task. In both *tactile & vis* blocks, they performed the tactile detection task while the target LED was briefly illuminated once during the trial; yet, only during the reaching block, this additional illumination provided additional reach-relevant information. In both *tactile & visDis* blocks, participants performed the tactile detection task while the target LED flashed either once or twice during the trial, and participants had to also discriminate whether they saw one or two flashes, i.e. they performed both the tactile detection task and the visual discrimination task. In the *tactile & visDis* reaching block, the visual signals did not only provide information about the target position, but were also relevant for the perceptual task.

In addition, a *control visDis* condition was conducted, in which participants only performed the visual discrimination task without the additional tactile detection task and without reaching. This resulted in a total of seven blocks per participant. In all blocks, participants were instructed to always fixate the target LED, even while this was switched off. The order of the 4 conditions was randomized across participants, but each participant performed either the reaching or the baseline block first and this order was held constant within participants and across conditions. A schematic depiction of the set-up and the timeline is shown in Fig 1 and a summary of the sensory signals presented in each reaching block is provided in Table 1.

**Table 1:** Summary of sensory information presented in each of the seven blocks. In all conditions, except the *visDis* baseline, a tactile stimulus is presented. This stimulus is accompanied with a single visual flash in the *tactile & vis* reaching block, and with either a single or a double visual flash to be discriminated in the *tactile & visDis* reaching block.

	Tactile input	Visual input Reach-relevant	Visual input Perception-relevant
*tactile* baseline	**X**		
*tactile* reaching	**X**		
			
*tactile & vis* baseline	**X**		
*tactile & vis reaching*	**X**	**X**	
			
*tactile & visDis* baseline	**X**		
*tactile & visDis reaching*	**X**	**X**	**X**
			
*visDis* baseline			**X**

In the *tactile* reaching block, each trial started with the two LEDs being illuminated (Fig 1): the starting LED indicated the start position of the reaching movement and the target LED indicated the reach goal. Participants were instructed to fixate the target LED throughout the trial. At the start of each trial, the starting and the target LEDs were illuminated for 1000 ms and 500 ms after the LEDs were switched off a 50 ms tone was presented prompting participants to reach to the target. We presented the vibrotactile stimulus on the moving index finger right after reach onset, as determined by lifting the finger off the touchscreen. Participants were told that they may or may not feel a tactile stimulus, but they received no information about the amount of trials with or without stimulation. Once participants finished their reach, confirmed by a short beep, a second 50 ms tone instructed them to indicate during a 1500 ms interval whether they had felt a stimulus or not by a left hand button press. In the reaching block of each condition, we presented six trials for each of the 15 stimulus intensities and 18 catch trials without stimulation (16.7%), resulting in 108 trials per block.

In the *tactile* baseline block, participants performed the tactile detection task while their hands remained still throughout the trial. Their right hand was resting on the frame of the touchscreen. To keep the visual input consistent with the reaching block, the target LED was switched on for 500 ms and was then switched off, however it was not relevant for the tactile detection task. The vibrotactile stimuli had the same characteristics as in the reaching blocks and were presented 250 ms after the LED was switched off. Participants had to give their detection response 250 ms later, during a 1000 ms response interval. Each baseline block of every condition comprised 108 trials (6 repetitions of the 15 intensities and 18 catch-trials).

The *tactile & vis* reaching and baseline blocks were identical to the *tactile* blocks, except that additional visual information was provided simultaneously with the presentation of the tactile stimulus. More specifically, together with the onset of the tactile stimulus, the target LED was switched on for 70 ms. In the reaching block, this additional illumination of the LED was reach-relevant as it provided information about the target position.

The *tactile & visDis* reaching and baseline blocks were identical to the *tactile & vis* blocks, but now the target LED flashed either once for 70 ms (identical to the *tactile & vis* blocks) or twice (two flashes of 10 ms each, with an interval of 50 ms in between) simultaneously with the onset of the presentation of the tactile stimulus. After giving the tactile detection response during a 1500 ms interval, a tone prompted participants to indicate whether they saw one or two flashes during another 1500 ms interval. Because participants had to give two different responses in this condition, we slightly increased the duration of each response interval by 500 ms. Of the 108 trials, 54 trials involved a single flash and 54 trials a double flash, while the six repetitions of each tactile stimulus for each intensity were presented half with a single and the other half with a double flash.

To assess the baseline visual discrimination performance without the simultaneous processing of the vibrotactile stimuli and the reaching movement, participants also performed the *control visDis* condition. Here, the target LED was illuminated for 500 ms, then switched off for 250 ms, and then flashed either once or twice as in the *tactile & visDis* condition. After 250 ms, a tone prompted participants to indicate the number of flashes, as in the *tactile & visDis* condition. Each flash was presented 54 times, resulting in 108 trials.

At the end of each experimental session one calibration trial was presented for each combination of starting and target LED. During the calibration, participants reached from the illuminated starting LED to the corresponding illuminated target LED.

### Data analysis

The first step in our analysis was to confirm that our manipulation was effective and the additional visual signals indeed influenced the reaching movement, and thus were processed. To do so, we analysed kinematic measures related to the target position, such as the endpoint accuracy and precision. Movement onset was determined as the first frame of the data collection: kinematic data collection started once participants released the finger from the touchscreen after the starting tone. The movement end was determined on the basis of the Multiple Sources of Information method [26]. The likelihood of a frame being the end of the movement increased with lower vertical positions, with lower movement tangential velocities, with higher tangential accelerations, and it had to be after the time of the movement onset. The frame at which the product of these likelihoods was the largest was considered to be the reach endpoint of the respective trial. We calculated the endpoint error as the three-dimensional Euclidean distance between the endpoint of the trial and the calibrated target position. The endpoint error is a measure that reflects endpoint accuracy. For every condition, the endpoint error was calculated for each trial, averaged across target positions and then across participants. We also calculated the endpoint variability along the movement direction as the standard deviation of the endpoint distribution along that direction. The endpoint variability reflects the precision of the reaching endpoints. For each condition, the endpoint variability was calculated and averaged for each target position, and then averaged across participants. To examine the effect of the additional reach-relevant visual information on endpoint error and variability we compared these variables between the *tactile* and the *tactile & vis* conditions. To investigate the effects of the perception-relevant visual signals, we compared endpoint error and variability between the *tactile & vis* and *tactile & visDis* conditions.

To examine tactile perception, we calculated for each individual participant the proportion of detected tactile stimuli for each baseline and reaching block of each condition. We then fitted these data of each participant to a logistic function using the maximum-likelihood estimation with the function *psignifit* in Matlab [27]. With this procedure we estimated the detection threshold and the detection precision. The detection threshold was defined as the 50% point of the psychometric function and the detection precision was defined as the difference in stimulus intensity between the 50% and the 84% of the psychometric function. To quantify the impact of reaching on tactile perception we subtracted each participant’s detection threshold and precision obtained in each baseline block from the respective values obtained in each respective reaching block. We consider the resulting threshold and precision differences (*detection*_*diff*_ and *precision*_*diff*_) to represent the strength of tactile modulation for each participant. The more positive the values of the *detection*_*diff*_ and *precision*_*diff*_ are, the stronger is the tactile suppression and the uncertainty of detection, suggesting a worse performance compared to the baseline block. For each condition, the baseline detection threshold and precision, as well as the threshold_diff_ and precision_diff_ were calculated separately for each participant and then averaged across participants.

Participants whose estimated detection thresholds were outside the range of stimulation in any of the blocks were excluded from all further analyses. More specifically, five participants had estimated detection thresholds greater than the strongest stimulus intensity in at least one reaching condition, while another two participants had estimated thresholds below zero (due to a high rate of false alarms) in at least one of their baseline blocks. Participants whose movement onsets were not successfully registered due to technical problems with the touch screen in at least half of the trials of at least one reaching block were also excluded from all further analyses. Based on this criterion, two additional participants were excluded. In total, from the original sample of 22 participants, nine were excluded from the final analyses.

To exclude the possibility that potential effects on tactile perception were due to the presentation of the additional visual signal, and not due to its consideration for reaching, we tested for differences in the baseline threshold and precision between the *tactile* and *tactile & vis* conditions. To further evaluate the effect of the reach-relevant visual signals on tactile perception during reaching, we examined the differences in threshold_diff_ and precision_diff_ between the *tactile* and *tactile & vis* conditions.

In a next step, we investigated the effects of the perception-relevant visual signals on tactile perception. In the *tactile & visDis* reaching block, the number of flashes was not relevant for the reach but was relevant for the visual discrimination task. We first evaluated the participants’ visual discrimination performance by calculating the proportion of trials in which participants gave a correct response about the number of flashes. For each of the blocks that involved the visual discrimination task (*control visDis*; *tactile & visDis* baseline; *tactile & visDis* reaching), we first calculated the proportion of correct responses for each participant and then averaged across participants. Visual discrimination performance was statistically tested against chance (50% correct). We then investigated how the processing of perception-relevant signals influenced the baseline tactile perception. To do so, we tested whether baseline detection threshold and precision differed between the *tactile & vis* and *tactile & visDis* conditions. To examine the effect of these signals on tactile perception during reaching, we then tested for differences in threshold_diff_ and precision_diff_ between the *tactile & vis* and *tactile & visDis* conditions. Finally, we investigated the relationship between the strength of suppression and the visual discrimination performance in the *tactile & visDis* condition. We assumed that if tactile suppression serves to free capacities for perception-relevant visual signals, then better visual discrimination performance should be related to stronger tactile suppression.

All comparisons between conditions were performed using two-tailed t-tests and relationships between variables were examined with bivariate correlations (p < 0.05). Possible null effects were examined with Bayes factors (BF) that quantified the likelihood of the null hypothesis being true compared to the alternative. The computed BF were interpreted according to the guidelines proposed by Jeffreys [28].

## Results

### Effects of additional visual signals on movement control

**Fig 2:**
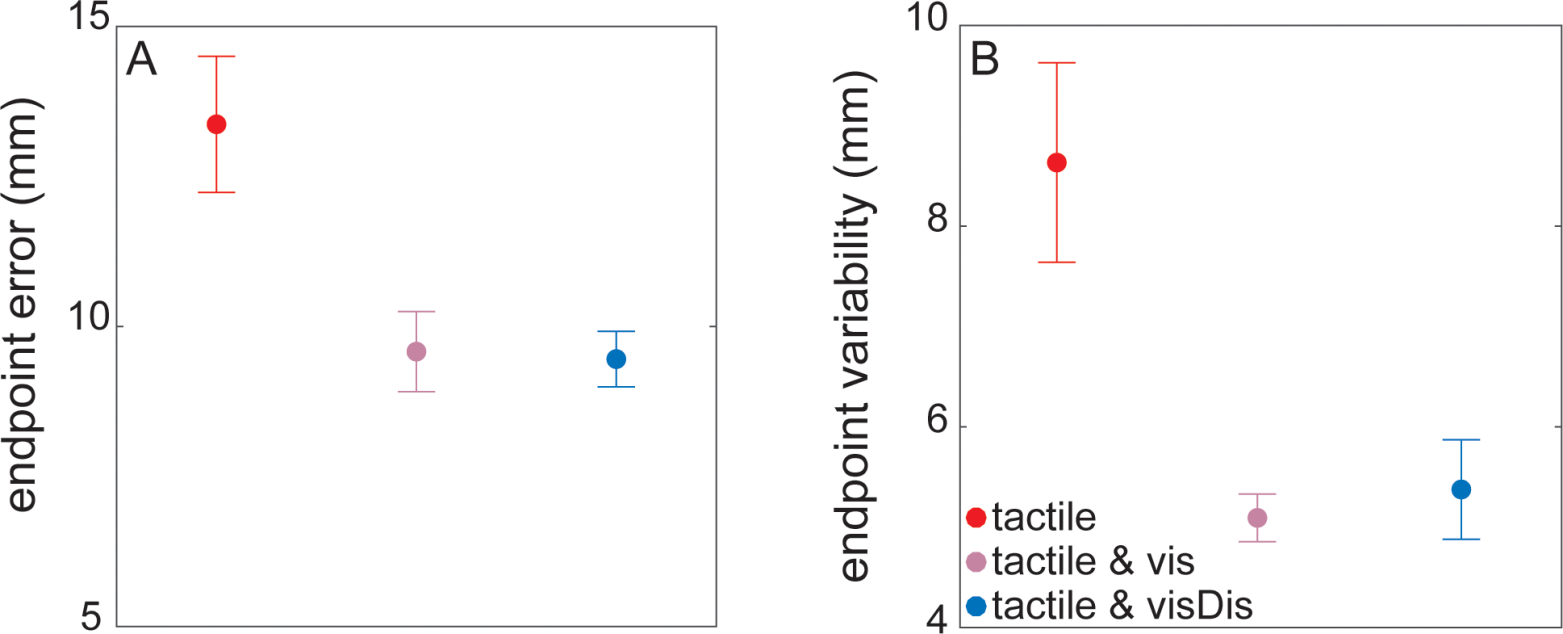
**Effects of the visual signals on (A) endpoint error, and (B) endpoint variability.** Error bars show the standard error of the participants’ mean.

We first examined the kinematics to confirm that the additional visual signals were indeed used for the guidance of the reaching movement (Fig 2). The additional illumination of the target resulted in lower endpoint errors (*t*_*24*_ = 2.88, *p* = 0.008) and lower variability (*t*_*24*_ = 3.46, *p* = 0.002) in the *tactile & vis* compared to the *tactile* condition. As expected, endpoint errors and variability were similar between the *tactile & vis* and *tactile & visDis* conditions (endpoint error: *t*_*24*_ = 0.15, *p* = 0.87; endpoint variability: *t*_*24*_ = 0.51, *p* = 0.61). These results demonstrate that participants did process the additional visual signals leading to improved movement performance.

### Tactile perception in baseline and reaching

**Fig 3:**
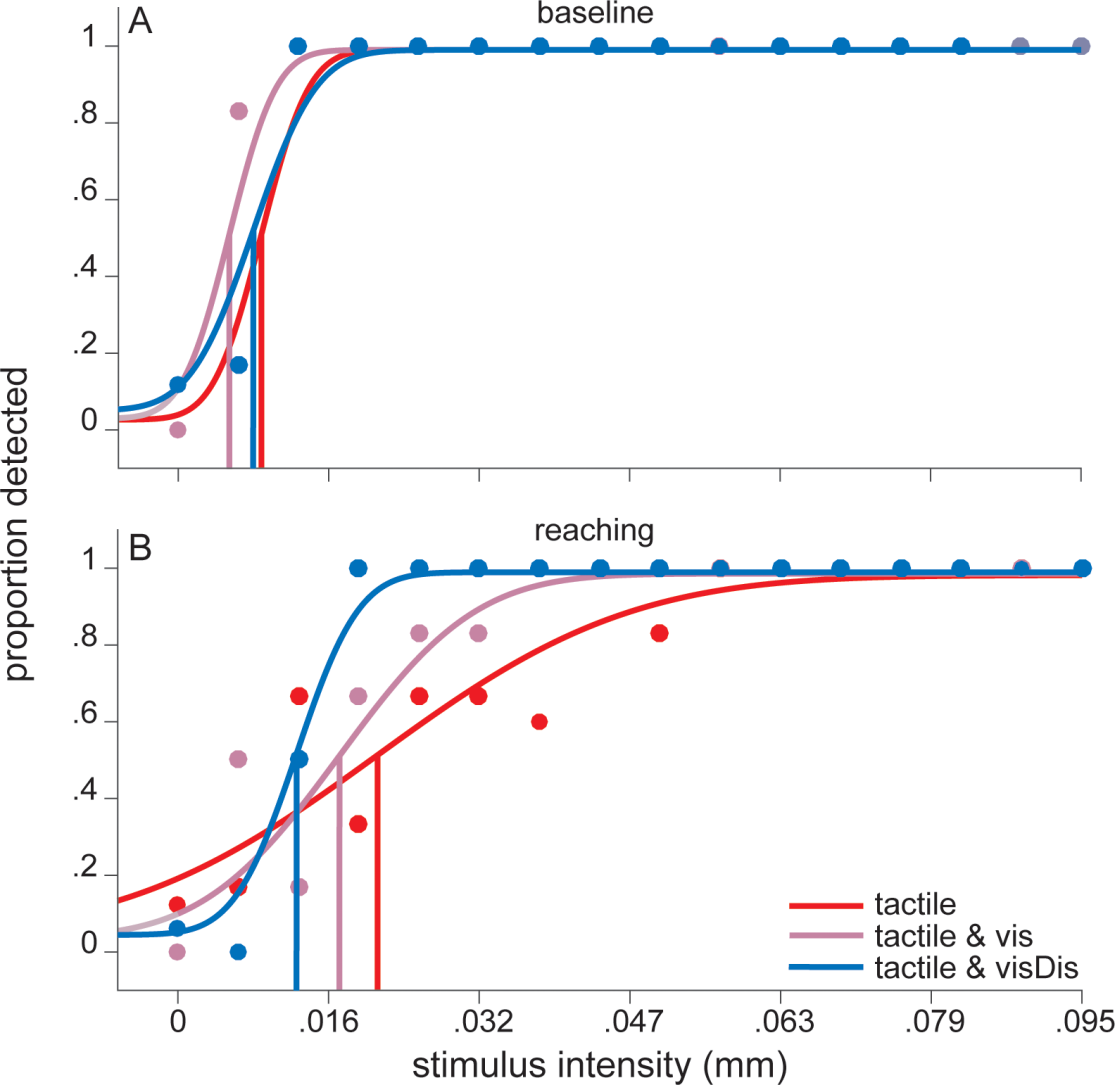
**Psychometric functions of a representative participant for (A) the baseline and (B) the reaching blocks of the three conditions.** The detection thresholds were low in the baseline and, as expected, increased during movement. For this participant, the precision of detection during reaching was poorer in the *tactile* condition better in the *tactile & vis* and best in the *tactile & visDis* condition. Note that for some stimulus intensities only one data point is evident because data points often overlap.

Psychometric functions of a representative participant are depicted in Fig 3. Detection thresholds were generally low in the baseline and clearly increased during reaching.

**Fig 4:**
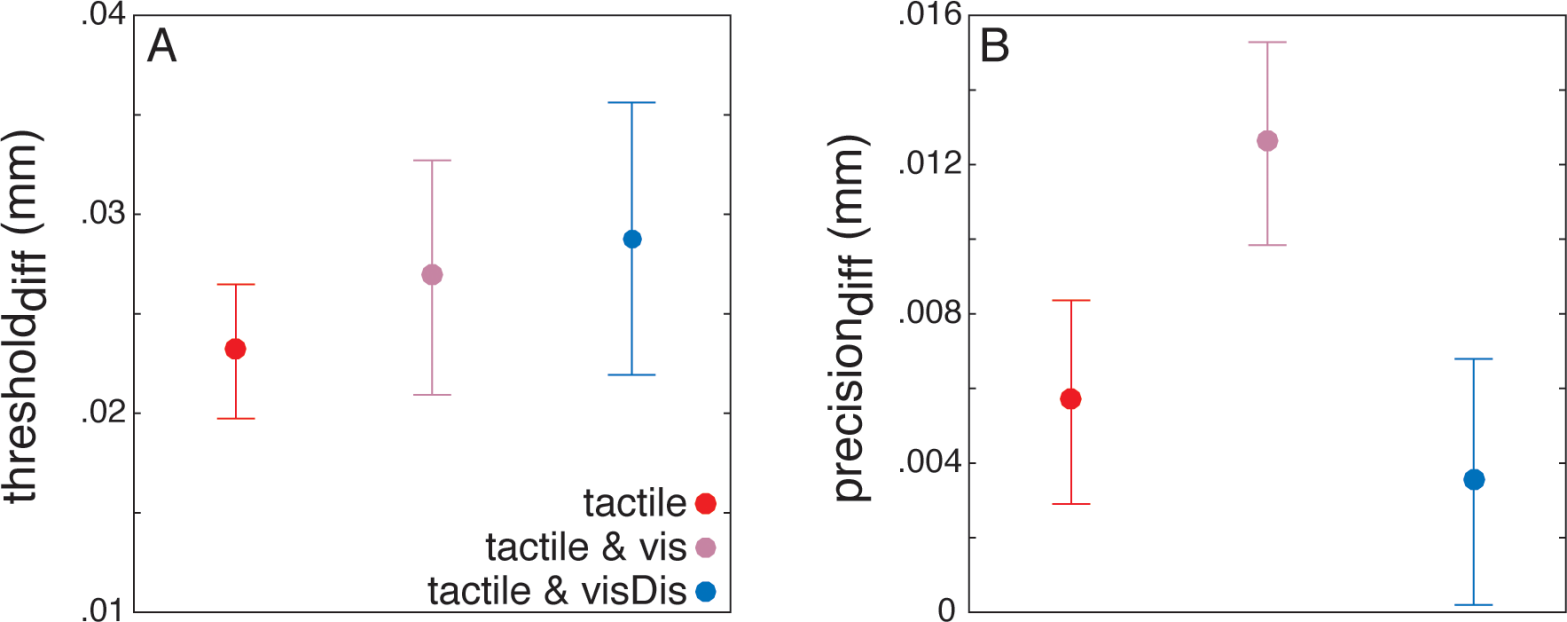
**The modulation of (A) detection thresholds, and (B) detection precision in each of the three reaching blocks with respect to the baseline.** Higher values in **A** and **B** indicate poorer accuracy and poorer precision of the tactile stimulus’ detectability, respectively. Error bars show the standard error of the participants’ mean.

The detection thresholds in all three baseline blocks was 0.01 ± 0.01 mm (mean ± standard error of the mean). As expected, detection thresholds increased during reaching compared to baseline in the *tactile* (*t*_*24*_ = 6.85, *p* < 0.001), *tactile & vis* (*t*_*24*_ = 4.55, *p* < 0.001), and *tactile & visDis* (*t*_*24*_ = 5.01, *p* < 0.001) conditions (Fig 4A). Tactile stimuli were thus clearly suppressed during movement. The detection precision was 0.005 ± 0.009 mm, 0.012 ± 0.009 mm, and 0.003 ± 0.012 mm for the *tactile*, *tactile & vis*, and *tactile & visDis* reaching blocks, respectively. The detection precision deteriorated in the *tactile* (*t*_*24*_ = 2.06, *p* < 0.049) and the *tactile & vis* (*t*_*24*_ = 4.61, *p* < 0.001) but not in the *tactile & visDis* condition (*t*_*24*_ = 1.06, *p* < 0.29; Fig 4B).

### Processing reach-relevant visual signals

We examined whether tactile suppression was stronger to facilitate processing the visual signals. In this case, we would expect stronger suppression when reach-relevant visual signals were processed, i.e., in the *tactile & vis* than the *tactile* condition. Despite the fact that the threshold_diff_ and the precision_diff_ were descriptively higher in the *tactile & vis* than the *tactile* condition, they did not significantly differ (threshold_diff_: *t*_*24*_ = -0.54, *p* = 0.58; BF = 0.39; precision_diff_: *t*_*24*_ = -1.79, *p* = 0.08; BF = 1.14).

### Processing perception-relevant visual signals

We next examined whether tactile suppression is stronger when additional perception-relevant visual signals need to be processed. We first tested participants’ performance in the visual discrimination task alone and when combined with other tasks: visual discrimination performance was well above chance in all conditions ranging on average from 69% to 78% correct (*visDis*: *t*_*24*_ = 4.39, *p* < 0.001; *tactile & visDis* baseline: *t*_*24*_ = 5.65, *p* < 0.001; *tactile & visDis* reaching: *t*_*24*_ = 3.45, *p* = 0.004). Thus, participants did process these perception-relevant visual signals irrespective of whether an additional tactile and/or motor task was simultaneously performed.

We hypothesized that tactile suppression would be stronger in the *tactile & visDis* than *tactile & vis* condition to facilitate the processing of signals related to the visual discrimination task. However, we found no differences in the threshold_diff_ between the *tactile & vis* and *tactile & visDis* conditions (*t*_*24*_ = -0.07, *p* = 0.94, BF = 0.28; Fig 4A), but the precision_diff_ deteriorated less in the *tactile & visDis* than the *tactile & vis* condition (*t*_*24*_ = 2.10, *p* = 0.044). In the *tactile & visDis* reaching block, the strength of suppression was not related to the performance in the visual discrimination task (*r* = -0.24, *p* = 0.43).

### Relationship between tactile suppression and movement kinematics

**Fig 5:**
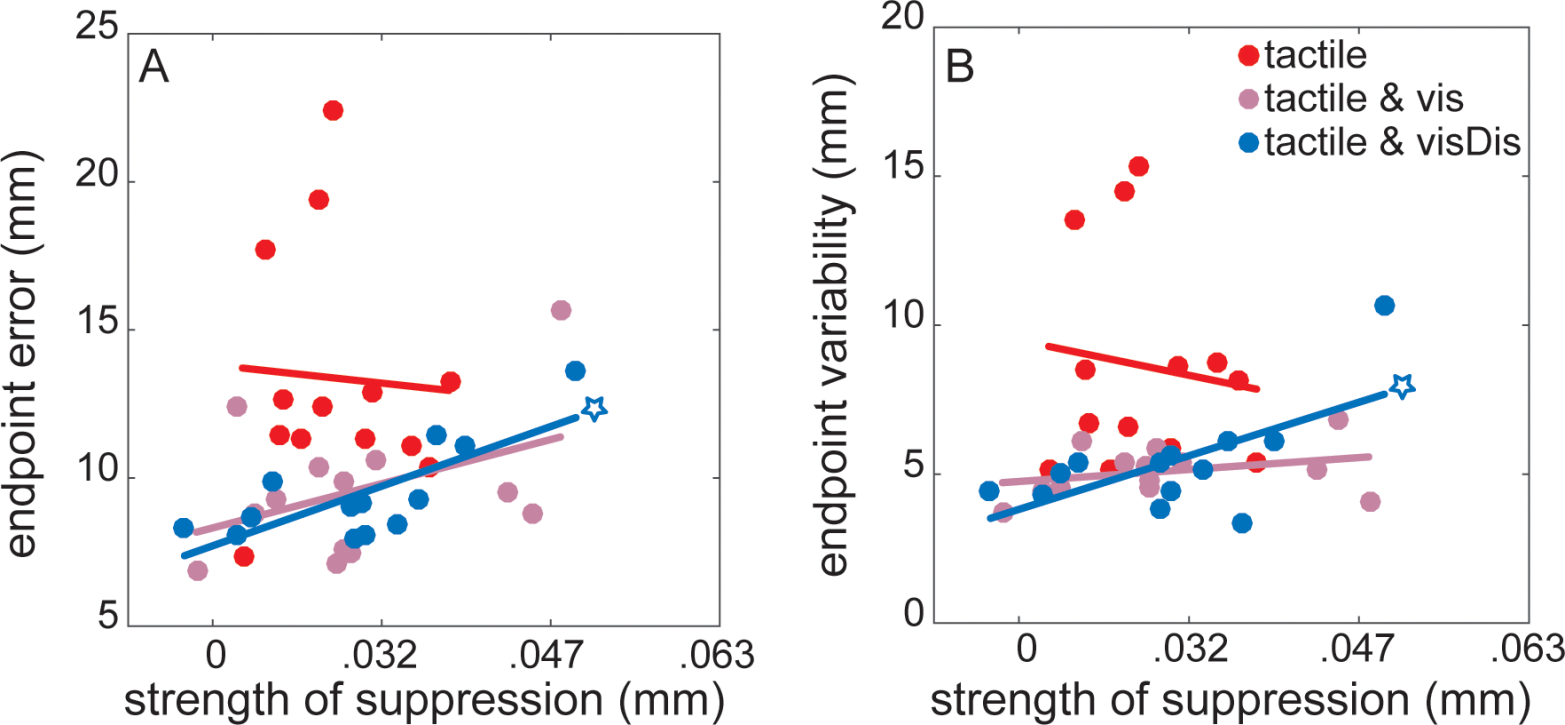
**Correlation between the strength of tactile suppression and the endpoint (A) error, and (B) variability for each condition.** Stars represent statistically significant correlations. Circles represent individual participants.

Lastly, we examined whether tactile suppression may release resources for improved movement control, i.e., for successfully guiding the reaching movement. In this case, we expected lower endpoint errors and lower endpoint variability with stronger tactile suppression. Although we found significant correlations between the strength of suppression and the endpoint error and variability in the *tactile & visDis* condition, the effect was in the opposite direction than expected: Participants who suppressed stronger showed greater endpoint errors (*r* = 0.76, *p* < 0.001; Fig 5A) and greater endpoint variability (*r* = 0.63, *p* = 0.02; Fig 5B) when simultaneously performing the visual discrimination task. These relationships did not reach statistical significance in the *tactile* (endpoint error: *r* = -0.05, *p* = 0.85; endpoint variability: *r* = -0.12, *p* = 0.68) and *tactile & vis* conditions (endpoint error: *r* = 0.41, *p* = 0.15; endpoint variability: *r* = 0.31, *p* = 0.29).

## Discussion

The purpose of this study was to examine the hypothesis that tactile suppression on a moving limb may occur to facilitate the processing of relevant sensory stimuli occurring elsewhere (cf., [1, 16]). We have recently shown that tactile suppression on a moving hand is stronger when reach-relevant somatosensory signals from another body part need to be processed [18]. Here we investigated whether tactile suppression is stronger when reach-relevant or perception-relevant visual signals need to be processed. We asked participants to detect a tactile stimulus on their moving hand presented while reaching to a previously illuminated LED in darkness either while the LED was off (*tactile*), or while it briefly flashed during reaching (*tactile & vis*), providing reach-relevant visual signals about the target position. If suppression occurs to facilitate processing these reach-relevant visual signals, we expected stronger suppression when such signals are provided. We also examined whether suppression is stronger when additional perception-relevant visual signals had to be processed: the target LED flashed either once or twice during reaching and participants had to discriminate the number of flashes besides performing the tactile detection task (*tactile & visDis*). If suppression occurs to facilitate processing perception-relevant visual signals, we expected stronger suppression when the visual discrimination task has to be performed.

We first showed that tactile detection thresholds in all reaching blocks were increased compared to the baseline blocks. This suggests that tactile perception during movement deteriorated and this is in line with previous work [9, 17]. After establishing this, we examined whether the illumination of the target in the *tactile & vis* and the *tactile & visDis* conditions improved endpoint accuracy and precision, and was thus processed as reach-relevant information. This was clearly the case (Fig 2). This also suggests that the additional visual discrimination task did not require processing demands that deteriorate movement guidance.

Given that the additional visual input was considered for the reach, we further investigated whether tactile suppression was stronger in the *tactile & vis* than the *tactile* condition when additional reach-relevant visual signals had to be processed. Our results show that such processing did not worsen (increase) tactile detection thresholds or detection precision. The similar tactile modulation between the *tactile* and *tactile & vis* conditions might have been caused due to the visual stimulus being presented early in the movement. During goal-directed hand movement, the movement is adjusted towards the target late in the movement, i.e. during the deceleration phase [29, 30]. Thus, the additional visual information might have been more important for the reach at a later stage of the movement. For this reason, we conducted an additional control experiment (not mention in the Results). Only the *tactile* and *tactile & vis* conditions were presented with the only difference from the main experiment being that both the tactile and the visual stimulus were now presented 200 ms after movement onset, instead of right after movement onset. Participants’ reaching movements clearly benefited from the additional target illumination with their endpoints being more precise (*t*_*24*_ = 4.01, *p* < 0.001) in the *tactile & vis* condition, but again the tactile modulation between the two conditions was similar (*t*_*24*_ = 0.05, *p* = 0.95).

In a second step, we wanted to investigate whether processing perception-relevant visual signals influences tactile suppression. To this end, together with the tactile detection task, participants performed also a visual discrimination task in which they had to report whether the target LED flashed once or twice. Participants’ discrimination performance was significantly above chance but did not reach ceiling levels. Given that visual discrimination performance was stable between the baseline and the reaching blocks, the results are inconsistent with previous findings that show a compromise in task performance when a task is executed together with another task than in isolation [31, 32]. Importantly, the modulation of detection thresholds in the *tactile & visDis* condition was as strong as in the *tactile & vis* condition, while the detection precision was slightly better in the *tactile & visDis* than the *tactile & vis* condition. We do not have a clear explanation for the effect on detection precision, but both results contradict the hypothesis that tactile suppression facilitates processing of other relevant sensory input because in this case one would expect stronger suppression in the *tactile & visDis* condition. The lack of tactile modulation in this case supports the idea that sensory signals from different modalities can be processed simultaneously without any cost. This is likely due to independent processing resources for signals from different sensory modalities [33, 34]. Moreover, the visual discrimination task might have been not demanding enough to require additional processing resources to be freed through stronger tactile suppression. Yet, participants’ performance was ~70%, far from ceiling levels, which does not support this possibility, although we cannot confidently exclude the possibility that a more demanding visual discrimination task could have modulated tactile suppression.

We recently demonstrated that tactile suppression is stronger when reaching to the other unseen hand than to an external target [18]. Presumably, in that study, reach-relevant sensory signals from the target hand were important to successfully guide the movement during somatosensory reaching. Processing these signals was associated with increased tactile suppression on the moving hand, supporting the notion that tactile suppression may occur to release resources to process other sensory input [1, 16]. Here we found no evidence for stronger tactile suppression when processing additional reach-relevant visual input, provided in the *tactile & vis* and *tactile & visDis* reaching blocks (illumination of the target LED). This may be due to differences in processing visual and somatosensory targets during reaching. For instance, reaching to remembered proprioceptive targets is substantially less accurate and precise than reaching to visual targets [35–37]. Therefore, somatosensory signals from the unseen target hand may also need more processing resources than visual signals about the target position. At this point, it may be noteworthy that from our original sample, five participants were excluded because their detection thresholds were beyond the range of stimulation in at least one of the reaching blocks. Four of these five participants showed such high thresholds in at least one of the reaching blocks with additional visual information (*tactile & vis*, *tactile & visDis*), which may have possibly limited a further modulation of suppression in these conditions.

Lastly, we examined whether tactile suppression contributes to movement control. We expected that stronger suppression might be related to more accurate or precise reaching, but found no systematic relationship between the strength of suppression and the guidance of the reaching movement. However, when visual information was available, there was a tendency for participants who suppressed stronger to also show increased endpoint errors and increased endpoint variability. This reached significance levels only in the *tactile & visDis* condition, though. Thus, it may be the case that the perceptual processing demands imposed by the visual discrimination task led to a deterioration of movement guidance. It is unlikely that the stronger suppression itself led to the poorer reaching guidance, because we did not observe such a relationship in the *tactile* reaching block. Yet, despite the gain in movement guidance through the visual signals, as shown in increased endpoint accuracy and precision, reduced tactile sensitivity on the moving hand seems to lead to poorer movement control. This is in line with previous studies [38, 39], but may only happen in the presence of visual information that require processing capacities unrelated to the movement itself.

In the conditions were we presented both visual and tactile signals, these signals were presented simultaneously (*tactile & vis*, *tactile & visDis*). It is known that visual information influences the perception of redundant tactile signals but not vice versa [40]. Although here the visual and the tactile signals were not redundant, we cannot exclude the possibility of some interactions between the sensory modalities. Yet, it is important to note that all our measures are relative to a respective baseline; potential influences of the simultaneous presentation of the sensory signals would likely be washed out from our relative measure. Moreover, even in the baseline, the simultaneous presentation of the visual and tactile signals did not influence detection thresholds compared to the tactile condition.

In this study we provide no support for the hypothesis that tactile suppression frees capacities for processing other relevant visual signals during movement execution. This questions the generality of the assumption that underlies the principles of tactile suppression. Our results further suggest that signals from different sensory modalities can be processed at no compromise.
